# Mechanism of Melatonin in Alleviating Aluminum Toxicity in Plants: A Review

**DOI:** 10.3390/biology14101316

**Published:** 2025-09-23

**Authors:** Feige Wang, Xiaoli Li, Can Chen, Le Zhao, Yunmin Wei

**Affiliations:** 1 Key Laboratory of Plant Genetics and Molecular Breeding, Zhoukou Normal University, Zhoukou 466001, China; wangfeige1108@163.com (F.W.); xiaoli890107@163.com (X.L.); chenc02@126.com (C.C.); 2 Henan Plant Gene and Molecular Breeding Engineering Research Center, Zhoukou Normal University, Zhoukou 466001, China; 3 School of Pharmacy, Henan University of Chinese Medicine, Zhengzhou 450046, China; 4 Henan International Joint Laboratory of Translational Biology, Zhoukou Normal University, Zhoukou 466001, China

**Keywords:** aluminum toxicity, acidic soil, melatonin, Al sequestration, cell wall modification, organic acid anion exudation, antioxidant defense systems

## Abstract

Aluminum (Al) toxicity is a major limiting factor for plant growth and development in acidic soils. Melatonin, a plant growth regulator and signaling molecule, enhances resistance to multiple stresses. Recent studies show that melatonin alleviates Al toxicity through several complementary mechanisms. Here, we first outline the physiological and molecular impacts of Al stress and the external and internal strategies plants use to cope with it. We then summarize melatonin biosynthesis and its broader roles in stress adaptation. We focus on recent advances in melatonin-mediated mitigation of Al toxicity, highlighting four principal mechanisms: (i) the activation of antioxidant defense systems, (ii) the stimulation of organic acid anion exudation that chelates Al in the rhizosphere, (iii) the modification of cell wall composition to reduce Al binding sites, and (iv) the promotion of intracellular Al sequestration. We also discuss the crosstalk between melatonin and nitric oxide, as well as interactions with phytohormone signaling. Collectively, this review comprehensively synthesizes the current understanding regarding the role of melatonin in alleviating Al toxicity in plants, offering a promising strategy for crop production in acidic environments.

## Simple Summary

A major global challenge in agriculture is aluminum (Al) toxicity in acidic soils, which severely restricts crop growth and productivity. This review explores how melatonin, a natural plant compound, can mitigate Al-induced stress. We elucidate the mechanisms of Al phytotoxicity and demonstrate the protective role of melatonin in enhancing plant resilience. Melatonin assists plants in several ways, including helping them remove or safely store harmful aluminum. Understanding these mechanisms will facilitate the development of effective management approaches to improve crop yields and food security in difficult acidic soil environments.

## 1. Introduction

Aluminum (Al), the third most abundant element in the terrestrial crust, primarily exists as aluminosilicates and oxides that remain non-toxic to plants in neutral or slightly acidic soils [1]. However, when soil pH falls below 5.5, aluminosilicate minerals undergo dissolution, releasing soluble Al species including Al(OH)_2_^+^, AlOH^2+^, and the highly phytotoxic Al^3+^ ions. These solubilized Al forms, particularly Al^3+^, exhibit pronounced toxicity toward the root apex, rapidly inhibiting root growth and consequently reducing plant capacity for water and nutrient absorption. This cascade of effects leads to nutritional deficiencies, physiological drought stress, and severe reductions in plant productivity [2,3]. Soil acidification occurs naturally through mineral weathering and base cation leaching but has been dramatically accelerated by intensive ammonium-based nitrogen fertilization, acid precipitation, and industrial emissions [4,5,6]. Currently, acidic soils constitute nearly 50% of the global potentially arable land; the widespread occurrence of Al toxicity as the primary limiting factor in acidic soils has made the development of effective mitigation strategies a critical priority for global food security [7,8,9].

Plants have evolved various strategies to cope with Al stress, which can be broadly categorized into two main approaches: avoidance, which prevents Al uptake and cell entry, and internal detoxification, which promotes Al vacuolar sequestration or enhances antioxidant capacity [10,11]. Recent discoveries in *Arabidopsis* have identified Al receptors and signal transduction modules governing transport and detoxification pathways through Sensitive to Proton Toxicity 1 (STOP1)-dependent transcriptional activation [12,13,14,15,16]. Although progress has been made in understanding how plants respond to Al stress, the molecular mechanisms underlying plant Al tolerance remain to be fully explored. Therefore, cultivating Al-resistant varieties adapted to acidic soils will be a long and challenging process. Fortunately, the application of biostimulants provides a viable and promising approach to addressing Al toxicity while maintaining agricultural production [17]. Melatonin (N-acetyl-5-methoxytryptamine), widely present in plants, significantly influences numerous physiological and biochemical processes [18,19]. Previous research has demonstrated the involvement of melatonin in multiple plant developmental processes, including root system architecture development, leaf senescence delay, photosynthesis enhancement, and circadian rhythm regulation [20]. Of particular relevance is the fundamental role of melatonin in root development through precise orchestration of cell division, elongation, and differentiation processes [21,22]. This is achieved through maintenance of optimal reactive oxygen species (ROS) homeostasis, particularly for hydrogen peroxide and superoxide anions, which function as essential second messengers. These ROS establish critical oxidative gradients that delineate boundaries between stem cell maintenance and cell differentiation zones [23,24,25,26]. Beyond its developmental roles, substantial evidence indicates that both endogenous and exogenously applied melatonin enhance plant responses to numerous abiotic stresses, including extreme temperatures, excessive light, and salinity stress [27,28,29]. Given these multifaceted protective roles, melatonin has emerged as a promising candidate for mitigating Al toxicity in agricultural systems [30,31]. However, despite growing evidence supporting the effectiveness of melatonin, there remains a lack of comprehensive reviews synthesizing current knowledge. This article provides a thorough overview of recent advances in understanding the mechanisms by which melatonin alleviates Al toxicity in plants, with a focus on practical applications for maintaining crop production in acidic soils.

## 2. Al Toxicity in Plants: Damage and Adaptive Mechanisms

### 2.1. Effects of Al Toxicity on Plant Growth

Aluminum toxicity triggers a cascade of phytotoxic effects that severely compromise plant growth and development [32]. The threshold concentration of Al^3+^ that causes root damage varies between species, with sensitive plants exhibiting growth inhibition at 5–10 µM, whereas tolerant species can withstand concentrations exceeding 200 µM [33]. The root apical meristem, which orchestrates root growth and development, is particularly vulnerable. The transition zone between the meristem and elongation zone represents the most susceptible region [34,35]. Within this critical region, Al^3+^ bound to negatively charged cell wall components triggers rapid morphological alterations, including root tip swelling and thickening, abnormal lateral root initiation, peripheral tissue disruption, and the excessive vacuolation of cortical cells [36]. These structural changes occur within minutes to hours of Al exposure, with the timing varying based on species-specific tolerance mechanisms.

At the cellular level, Al toxicity operates through multiple interconnected pathways. Aluminum destabilizes plasma membrane function by binding to phospholipid bilayers, inhibiting H^+^-ATPase activity, and disrupting ion transport systems, leading to reduced uptake of essential nutrients, including nitrogen, phosphorus, potassium, calcium, magnesium, and micronutrients [37,38]. Moreover, Al exposure triggers oxidative stress through NADPH oxidase activation and mitochondrial dysfunction, leading to excessive ROS accumulation that induces lipid peroxidation, malondialdehyde generation, membrane damage, protein degradation, and ultimately programmed cell death [39]. At the molecular level, Al penetrates cells and binds to DNA phosphodiester backbones, altering conformation and causing fragmentation, chromosomal aberrations, and cell cycle disruption. Studies on DNA damage response gene mutants indicate that Al primarily induces double-strand breaks rather than single-strand breaks [40]. Additionally, Al destabilizes cytoskeletal components by disrupting microtubule and actin filament organization, potentially through direct interaction or indirect Ca^2+^ signaling disruption, further impairs cell division and growth [41]. Cellular damage cascades into system-wide physiological impairments, with compromised root function creating physiological drought conditions despite adequate soil moisture availability [42]. Photosynthetic capacity becomes significantly impaired through reduced chlorophyll content, disrupted photosystem II function, and decreased gas exchange parameters, ultimately leading to diminished CO_2_ assimilation and reduced productivity [43,44].

Aluminum toxicity in acidic soils rarely occurs alone; it typically coincides with other limiting factors, forming complex multi-stress environments with synergistic interactions [45]. The Al–drought stress interaction is particularly damaging, as both factors constrain root development through overlapping mechanisms that create negative feedback loops [46]. Aluminum-induced root damage reduces soil volume available for water extraction, while drought conditions concentrate Al^3+^ in the diminished soil solution, intensifying Al exposure [47]. Both stresses generate excessive ROS production, depleting shared antioxidant defense systems [48]. Moreover, phosphorus deficiency becomes particularly acute as Al forms insoluble Al-phosphorus complexes in soil [49]. This acidification simultaneously disrupts microbial community dynamics and mobilizes toxic heavy metals (notably cadmium and lead), with these effects intensifying at pH levels below 4.5 [50]. Therefore, understanding these complex stress interactions is essential for developing effective management strategies for acidic soil agriculture. Melatonin exhibits broad-spectrum protective mechanisms, including antioxidant activity, membrane stabilization, stress-responsive gene regulation, and hormonal coordination, making it particularly valuable for addressing the interconnected challenges in acidic soil environments [51].

### 2.2. Mechanisms of Al Tolerance in Plants

Plants have evolved sophisticated mechanisms to cope with Al stress, broadly categorized into avoidance and internal detoxification strategies [52,53]. The secretion of OAs from roots represents the most extensively characterized resistance mechanism [54]. This process operates through the chelation of toxic Al^3+^ ions, forming stable, non-toxic complexes that prevent Al from entering root cells [55]. Different species employ distinct OA strategies; for instance, the rubber tree (*Hevea brasiliensis*) primarily relies on malate and oxalate secretion, while the soybean (*Glycine max*), maize (*Zea mays*), and wheat (*Triticum aestivum*) predominantly secrete citrate [56,57]. In addition to OA secretion, cell wall modifications constitute another crucial avoidance mechanism. Pectin content, degree of methylesterification, and xyloglucan structure significantly influence Al^3+^ binding capacity. Both pectin demethylesterification and xyloglucan property changes affect the density of negative charges available for Al binding [58,59].

When Al penetrates avoidance barriers, plants activate internal detoxification mechanisms. The primary strategy involves compartmentalization of Al^3+^ away from metabolically active sites through vacuolar sequestration [60]. Specific transport proteins facilitate this process. In rice, the OsNRAT1 transports Al from the cell wall into the cytoplasm, followed by vacuolar sequestration via the tonoplast-localized ABC transporter OsALS1 [61,62]. Similar mechanisms operate in Al-accumulating species, such as buckwheat (*Fagopyrum esculentum*) [63] and hydrangea (*Hydrangea macrophylla*) [64], which complex Al with oxalate before vacuolar storage. Enhanced antioxidant capacity constitutes another crucial internal strategy. Aluminum-tolerant plants typically upregulate antioxidant enzymes, including superoxide dismutase (SOD), catalase (CAT), and peroxidases (POD), to neutralize ROS, protecting cellular components from oxidative damage [65,66].

## 3. Melatonin Biosynthesis and Its Role in Stress Responses in Plants

### 3.1. Melatonin Biosynthesis in Plants

Since the first demonstration of de novo melatonin biosynthesis in *Hypericum perforatum* seedlings [67], it has become clear that higher plants are true melatonin producers. Unlike the relatively straightforward animal pathway, plant melatonin synthesis exhibits remarkable complexity with at least four to six different enzymatic routes involving not only the classical enzymes tryptophan decarboxylase (TDC), tryptamine 5-hydroxylase (T5H), serotonin N-acetyltransferase (SNAT), and N-acetylserotonin methyltransferase (ASMT), but also alternative pathways utilizing tryptophan hydroxylase (TPH) and caffeic acid o-methyltransferase (COMT), creating a highly redundant biosynthetic network that allows continued melatonin production even when specific enzymes are compromised [51,68]. This network is further diversified by the species-specific expression patterns and subcellular partitioning across chloroplasts, mitochondria, and cytosol [51,69].

### 3.2. The Diverse Roles of Melatonin in Plant Stress Responses

Elucidating the biosynthetic pathway of melatonin and its regulatory roles in plant stress responses has become a focal point in stress resistance research [70,71]. Acting as a hub in the plant-hormone network, melatonin integrates signals that range from epigenetic remodeling to post-translational protein modification, coordinating multiple stress-responsive pathways [72]. Melatonin functions as a potent multifunctional stress protectant, significantly enhancing plant adaptability to diverse abiotic stresses [73,74,75]. In alkaline conditions, exogenous melatonin stimulates polyamine biosynthesis, enhances ROS-scavenging systems, and promotes growth and photosynthesis in *Malus hupehensis*, rice, and soybean [76,77,78]. In cold-stressed pepper seedlings, melatonin stabilizes the photosynthetic apparatus and adjusts pigment and osmolyte levels to preserve development [79]. During drought, melatonin boosts antioxidant activities, increases cell turgor and water-holding capacity, prevents stomatal closure to sustain light capture and CO_2_ fixation, and upregulates aquaporins for improved root water uptake [80,81,82,83,84]. In heavy-metal exposure, melatonin drives metal chelation, compartmentalization, and nutrient balance to alleviate cadmium and lead toxicity [85,86]. During heat stress, melatonin enhances tolerance by regulating antioxidant defense mechanisms and maintaining osmotic balance [87]. Under salinity, exogenous melatonin treatment stimulates endogenous melatonin biosynthesis while increasing sugars and proline accumulation, and optimizing K^+^/Na^+^ ratios [88,89,90]. These studies systematically reveal the diverse mechanisms through which melatonin acts as a central regulator of plant stress resistance.

## 4. Melatonin-Mediated Alleviation of Al Toxicity

Numerous studies have demonstrated the beneficial effects of melatonin in mitigating Al toxicity across various plant species (Table 1). Melatonin alleviates Al stress through multiple mechanisms operating at different cellular and physiological levels, including improving antioxidant enzyme activities, enhancing OA exudation, modifying cell wall composition, and facilitating vacuolar sequestration [91].

### 4.1. Melatonin-Mediated Enhancement of Antioxidative Defense Systems

Aluminum toxicity triggers extensive ROS generation as a primary stress response across plant species [99,101,102,103]. When exposed to Al, cellular homeostasis becomes severely disrupted through multiple interconnected pathways such as impaired electron transport chain functionality, enhanced NADPH oxidase activation at plasma membranes, and systematic deterioration of native antioxidant defense systems, thereby creating an amplified oxidative environment that intensifies Al phytotoxic effects [34,104]. Plants have evolutionarily developed sophisticated defensive strategies, especially the accumulation of enzymatic antioxidants, to counteract oxidative challenges [105]. Within this defense network, melatonin emerges as a remarkably versatile molecular guardian, demonstrating exceptional efficacy across diverse environmental stressors, including arsenic [106,107], cadmium [108,109], cold [110,111], copper [112,113], drought [114,115], iron [116], and salt stress [117,118]. It effectively neutralizes substantial amounts of ROS, enhances activities of various antioxidant enzymes, and regulates hydrogen peroxide accumulation [119]. Studies show that the exogenous melatonin significantly enhances CAT, POD, and SOD activities in soybean roots, mitigating Al-induced oxidative damage [120]. Similar reinforcement of redox buffering has been documented for alfalfa, rice, and wheat, accompanied by decreased cytosolic H_2_O_2_ concentrations that modify downstream signaling cascades [31,98,101]. Additionally, melatonin enhances the regeneration of ascorbate (AsA) and glutathione (GSH) under various abiotic stresses [121,122]. Excitingly, upregulation of melatonin biosynthetic genes significantly enhances antioxidant enzyme activities [72]. Overexpression of *SlCOMT* enhances melatonin synthesis, thereby enhancing the tolerance to salt–alkali stress in tomatoes [123]. Similarly, *ClCOMT1* overexpression in watermelon and *PtCOMT5* in citrus enhances stress through promoting the melatonin accumulation [124,125]. Moreover, plant growth-promoting bacteria capable of producing melatonin offer an effective strategy for alleviating Al toxicity. Similar findings have been observed in *Bacillus safensis* EH143, which mitigates salt and cadmium stress in soybean [126]. In summary, the synthesis and regulation of melatonin are effective strategies for plants to alleviate Al toxicity.

### 4.2. Melatonin Promotes OA Exudation and Chelation of Al

**Figure 1:**
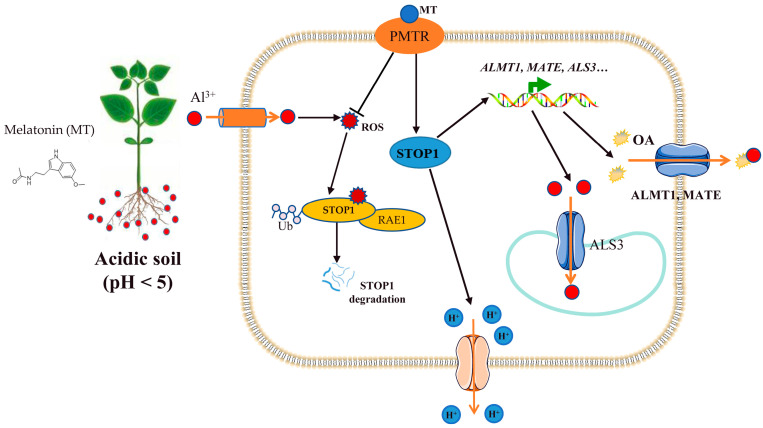
Schematic representation of melatonin-mediated alleviation of Al toxicity through OA secretion. MT application enhances antioxidant enzyme activities, eliminating ROS and stabilizing STOP1, which upregulates OA transporter genes. Increased OAs release from root apices into the rhizosphere, forming Al-OA complexes. Additionally, MT maintains proton (H^+^) homeostasis by promoting H^+^ efflux, contributing to cytoplasmic pH alkalization under Al stress. OAs, organic acid anions; MATE, multidrug and toxic compound extrusion; ROS, reactive oxygen species; STOP1, Sensitive to Proton Toxicity 1.

Root exudation of OAs represents a primary means of both external and internal Al detoxification, as these anions chelate Al^3+^ in the rhizosphere and block uptake [104]. Intriguingly, melatonin does not constitutively drive OA exudation but specifically amplifies this response under Al stress (Figure 1). In soybeans, exogenous melatonin sharply increases citrate and malate efflux only when roots are challenged with Al, indicating a stress-dependent signal mobilization of organic anion transport [95]. Similarly, melatonin priming elevates citrate concentration in rice root apexes, effectively barricading Al entry into the symplast [98].

At the transcriptional level, melatonin upregulates key OA transporter genes by enhancing the expression of the master regulator STOP1. In apple, melatonin elevates STOP1 transcript levels, which subsequently bind promoters of the OA transporter genes to boost citrate secretion and diminish root Al accumulation [97]. Recent studies show that H_2_O_2_-induced STOP1 degradation through interaction with F-box protein RAE1 adversely affects Al resistance [127]. Conversely, decreasing H_2_O_2_ content blocks STOP1 oxidation and increases stability, demonstrating that melatonin can promote OA secretion by decreasing ROS. However, species-specific differences exist; melatonin decreases ROS in both *Arabidopsis* and wheat under Al stress but only enhances OA secretion in *Arabidopsis* [92,101]. Moreover, the Al receptor (ALR1) in *Arabidopsis* interacts with STOP1 through ROS; whether an analogous mechanism operates in other species remains unclear. Collectively, current evidence supports a model in which melatonin enhances OA secretion through ROS buffering and STOP1 stabilization; however, the precise regulatory modules differ between plant species and warrant further investigation.

### 4.3. Melatonin Reduces Cell Wall Al Accumulation and Alleviates Al Toxicity

**Figure 2:**
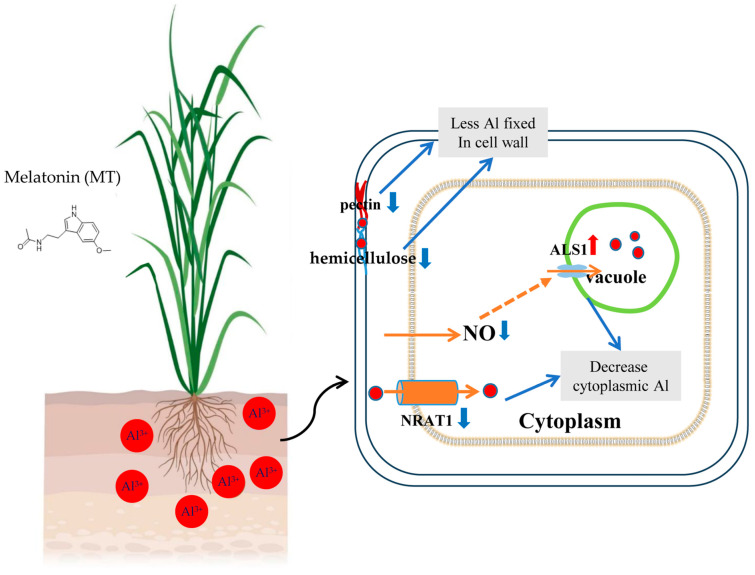
Schematic representation of melatonin-mediated alleviation of Al toxicity by reducing CW Al accumulation and vacuolar sequestration. MT suppresses Al-induced pectin methylesterase activity, maintaining a lower degree of pectin methylesterification and limiting the formation of negatively charged carboxyl groups that serve as binding sites for Al^3+^. MT attenuates Al-induced upregulation of CW biosynthetic and modifying genes as well as lignification processes, collectively reducing Al binding sites and decreasing CW permeability. Concurrently, MT enhances tonoplast proton pump activity and optimizes transporter balance, promoting Al^3+^ sequestration into vacuoles via ALS1 transporters while strategically limiting excessive cytosolic influx through NRAT1. These protective mechanisms appear to be orchestrated through MT-mediated modulation of nitric oxide (NO) content and associated signal transduction pathways.

Aluminum binding to the cell wall is a prerequisite for toxicity, and the root cell wall represents the first barrier encountered by Al^3+^ ions [42,128]. Under Al stress, the cell wall polysaccharide composition, especially pectin and hemicellulose, typically increases, providing numerous negatively charged carboxyl groups as Al^3+^ binding sites [103]. Accumulating evidence shows that exogenous melatonin markedly reduces wall-bound Al in crops like wheat and rice [98,101]. Melatonin exerts this effect through dual mechanisms involving pectin chemistry and gene expression. It suppresses the Al-induced pectin methylesterase activity, maintaining less degree of pectin methyl-esterification and reducing Al-binding sites [101]. Concurrently, melatonin downregulates Al-induced transcripts for cellulose synthases, xyloglucan endotransglucosylase/hydrolases, additional pectin methylesterases, and phenylpropanoid pathway enzymes responsible for lignin deposition [31]. By restricting the new binding site formation and decreasing overall cell wall permeability, melatonin ultimately reduces Al uptake and significantly alleviates growth inhibition in acidic soils (Figure 2).

Vacuolar sequestration is the centerpiece of internal Al detoxification, as tonoplast transporters rapidly shuttle Al^3+^ into the vacuole, lowering its cytosolic concentration and reactivity [129]. Recent findings highlight the role of melatonin in amplifying this pathway through integrated control of proton pumps, transporter genes, and metal-binding processes [97]. In the Al-stressed *Malus hupehensis* roots, exogenous melatonin enhances vacuolar Al^3+^ compartmentalization by promoting H^+^/Al^3+^ exchange through the coordinated regulation of MdSTOP1, MdNAC2, MdALS3, MdNHX2, and MdAHAs, increasing H^+^-ATPase activity and maintaining ion homeostasis [97]. Similarly, in rice, melatonin boosts Nrat1 expression at the plasma membrane to import Al^3+^ into the cytosol and enhances OsALS1 levels at the tonoplast for vacuolar sequestration, thus establishing a cytosol-to-vacuole Al^3+^ transport route [99]. In soybeans exposed to Al, melatonin application stimulates citrate production, elevates *GmIREG3* expression, and augments vacuolar Al buildup, suggesting that it bolsters compartmentalization by activating Al transporters while potentially integrating with other protective strategies to minimize disruptions to cellular metabolism. Moreover, Al exposure heightens *GmALS1* and *GmCDT3* expression in soybean roots, an effect amplified by melatonin. Conversely, while Al induces *GmNrat1* expression and elevates cytosolic Al, melatonin counters this by repressing *GmNrat1* and curbing cytosolic accumulation, suggesting the downregulation of the ART1-Nrat1 pathway to limit internal Al accumulation [96]. Extending beyond transporter dynamics, melatonin promotes the production of sulfur-containing chelators like glutathione and phytochelatins, which bind Al^3+^ into inert, non-toxic forms suitable for vacuolar deposition [130]. Furthermore, because Al^3+^ competes with Ca^2+^ and Mg^2+^ uptake, the capacity of melatonin to preserve calcium and magnesium homeostasis protects membranes and enzymes during Al stress. Together, proton-coupled Al sequestration, improved chelator availability, and balanced nutrient status constitute a concerted melatonin-dependent strategy that markedly enhances the internal detoxification in plants (Figure 2).

## 5. Synergistic Effects of Melatonin, NO, and Phytohormones on Plant Responses to Al Stress

### 5.1. Melatonin Interactions with Nitric Oxide in Al Stress Response

Nitric oxide (NO) serves as a widespread signaling molecule playing a crucial role in numerous physiological functions and environmental stresses in plants [131]. NO production is rapidly induced in root apex cells under Al stress, whereas excessive NO accumulation promotes nitrosative damage, lipid peroxidation, and programmed cell death [132]. Recent research has demonstrated that melatonin orchestrates sophisticated regulatory mechanisms to modulate this NO network at multiple cellular levels [133]. Plant defenses against stressors usually depend on the collaborative efforts of multiple signaling agents. Notably, melatonin and NO collaborate under various abiotic stresses [134]. For example, joint treatment with melatonin and NO curbs the uptake of cadmium and lead from soil by boosting the release of organic acid anions into the root zone, whereas NO production prompted by melatonin improves the resilience of tomato seedlings to high pH environments [135,136]. Comparable mechanisms contribute to mitigating Al poisoning as well.

Melatonin employs several distinct strategies to regulate NO homeostasis under Al stress. Direct scavenging represents a primary mechanism, effectively neutralizing excessive nitric oxide under cytotoxic conditions [137]. This proves particularly crucial during acute Al stress when NO accumulation can induce protein nitration and membrane damage [99]. Simultaneously, melatonin influences NO biosynthesis by modulating both nitric oxide synthase-like activity and nitrate reductase, both involved in Al-induced NO production and root growth inhibition [92]. Similar effects occur across diverse stress conditions, as exemplified in cadmium-stressed Chinese cabbage seedlings, where melatonin treatment effectively reduced NO synthesis enzyme activity and consequently mitigated oxidative damage [138].

Transcriptional regulation and post-translational modifications constitute another critical dimension of melatonin–NO crosstalk. In rice, melatonin-mediated NO regulation affects expression of key Al-responsive genes [99]. Specifically, melatonin coordinately regulated Al-related gene expression by downregulating *OsSTAR1/2* (hiding Al binding sites) and *OsNRAT1* (Al^3+^ absorption), while upregulating *OsALS1* (vacuolar sequestration), all of which collectively contributed to a reduction in cytoplasmic Al concentration. At the post-translational level, S-nitrosylation (NO conjugation to cysteine residues) represents a powerful regulatory mechanism that melatonin helps optimize [139]. Studies in tomato have demonstrated that melatonin confers saline–alkali tolerance by alleviating nitrosative damage and preventing S-nitrosylation of H^+^-ATPase 2 [90]. However, the specific mechanisms underlying melatonin-mediated S-nitrosylation regulation during Al stress remain unclear and warrant further investigation.

### 5.2. Melatonin Interactions with Phytohormones in Al Stress Response

Recent evidence indicates that the protective effects of melatonin against Al toxicity extend beyond direct detoxification to encompass sophisticated hormonal crosstalk that preserves root meristem integrity [140,141]. Melatonin modulates the biosynthesis and signaling of brassinosteroids (BRs), gibberellins (GAs), indole-3-acetic acid (IAA), and jasmonic acid (JA)—hormones central to meristem maintenance and cell cycle control [142,143,144,145]. Aluminum stress disrupts the root meristem cell division and arrests growth; melatonin protects this region by stabilizing hormone homeostasis [144]. At the auxin level, melatonin recalibrates IAA homeostasis and PIN-mediated polar auxin transport, thereby sustaining the auxin maxima and gradients essential for meristem organization and balanced stem cell activity under Al stress [22]. Concurrently, melatonin reinforces GA-mediated cellular elongation in the root transition zone by modulating gibberellin biosynthesis and signaling pathways [145]. This GA-driven response effectively counteracts Al-induced root growth inhibition while supporting developmental homeostasis. Furthermore, melatonin enhances BR signaling, which interfaces with growth-regulatory transcription factors to promote cell cycle progression and meristem maintenance [144,145]. Additionally, melatonin regulates JA metabolism by controlling lipoxygenase expression and modulating JA content, which influences lateral root development and stress responses [142]. Collectively, this sophisticated multilayered hormonal network synergizes with antioxidant and detoxification activities of melatonin to maintain the critical balance between cell division and differentiation, preserve stem cell niche integrity, and sustain root growth under Al stress conditions.

## 6. Conclusions

Melatonin emerges as a multifunctional protectant against Al toxicity in plants through multiple synergistic mechanisms. The protective pathways include ROS scavenging, organic acid anion efflux enhancement, cell wall modification, and optimization of vacuolar sequestration. The coordinated action of these mechanisms provides comprehensive protection against Al toxicity, preserving root growth and function. Future research should further explore the specific mechanisms of melatonin in alleviating Al toxicity, optimize application strategies, and develop genetic engineering technologies to enhance plant Al toxicity and productivity. In summary, melatonin is a promising, natural tool for mitigating Al toxicity and improving plant health and productivity in challenging acidic soil environments.

## Figures and Tables

**Table 1:** Melatonin alleviates Al toxicity in different plant species.

Plant Species	Al Concentration	Melatonin Concentration	Functions	Reference
*Arabidopsis thaliana*	100 µM	10 µM	Interferes with NO-mediated reduction in cell division cycle progression and the quiescent center cellular activity	[92]
*Brassica napus*	25 µM	50, 100 µM	Restricts the mobilization of Al into vacuoles and improves antioxidant potential	[93]
*Camelina sativa*	97 mg/L	50, 100, 200 µM	Increases the antioxidative enzyme activity	[30]
*Carya cathayensis*	50 µM	10 µM	Removes the excessive accumulation of ROS and decreases Al accumulation in roots	[94]
*Glycine max*	50 µM	100, 200 µM	Improves the activity of antioxidant enzymes and enhances the secretion of citrate	[95]
*Glycine max*	50, 100 µM	75 µM	Modification of cell wall and vacuolar compartmentalization of Al	[96]
*Malus hupehensis*	300 µM	1, 10 µM	Promotes Al^3+^ compartmentalization by enhancing vacuolar H^+^/Al^3+^ exchange	[97]
*Medicago sativa*	10 µM	5 µM	Reduces Al accumulation and restores redox homeostasis	[31]
*Oryza sativa*	150 µM	10, 50, 100 µM	Depresses the Al-induced synthesis of pectin and hemicellulose and increases citrate content	[98]
*Oryza sativa*	25 µM	20 μM	Promotes vacuolar compartmentation to lower cytoplasmic Al concentration	[99]
*Solanum lycopersicum*	148 µM	150 µM	Contributes to the reduction in Al translocation to the leaves	[100]
*Triticum aestivum*	30 µM	10 µM	Increases the activity of antioxidant enzymes and enhances the exclusion of Al from root apex	[101]
*Zea mays*	148 μM	50 µM	Modulates carbon and nitrogen metabolism, reestablishing redox homeostasis	[102]

## Data Availability

No new data were created or analyzed in this study.
